# Case Report: Lung squamous cell carcinoma mimicking recurrent aspergillosis in systemic lupus erythematosus

**DOI:** 10.3389/fmed.2026.1779338

**Published:** 2026-03-06

**Authors:** Jiawen Yu, Yuqi Tang, Sen Tian, Weijun Zhu, Qiaoding Dai

**Affiliations:** 1 The First Clinical Medical College, Zhejiang Chinese Medical University, Hangzhou, China; 2 Pathology Department, Taizhou Hospital of Zhejiang Province, Taizhou, China; 3 Department of Rheumatology and Immunology, The First Affiliated Hospital of Zhejiang Chinese Medical University, Hangzhou, China

**Keywords:** anchoring bias, brain metastases, case report, invasive pulmonary aspergillosis, lung squamous cell carcinoma, systemic lupus erythematosus

## Abstract

Distinguishing between life-threatening infection and malignancy in immunocompromised patients remains a major clinical challenge. Individuals with systemic lupus erythematosus (SLE) are at heightened risk for both invasive pulmonary aspergillosis (IPA) and lung cancer, and their coexistence can create a profound diagnostic dilemma. We report the case of a 66-year-old man with a history of SLE and prior IPA who presented with fever, headache, and progressive cognitive decline. Imaging revealed a cavitary lung lesion and multiple brain nodules, initially suggestive of intracranial IPA dissemination. Despite aggressive antifungal therapy, his condition rapidly worsened. A subsequent ^18^F-FDG PET-CT scan demonstrated intense hypermetabolism in the lung, brain, and multiple skeletal sites, shifting the diagnostic consideration toward metastatic malignancy. Lung biopsy ultimately confirmed poorly differentiated pulmonary squamous cell carcinoma (cT4N3M1c, Stage IV) with brain and bone metastases. This case highlights the diagnostic delays that can arise from anchoring bias toward prior infection and underscores the importance of recognizing “red flags” such as treatment failure, incorporating PET-CT to reassess disease biology, and relying on histopathological confirmation to overcome cognitive biases and diagnostic inertia in complex immunocompromised patients.

## 1 Introduction

Systemic lupus erythematosus (SLE) is a chronic autoimmune disease characterized by dysregulated immune activation, autoantibody production, and multi-organ involvement, predominantly affecting women of childbearing age (1). As of 2023, the global prevalence of SLE is estimated at approximately 3.4 million, with an incidence of around 5.14 per 100,000 individuals (2). Due to both intrinsic immune dysfunction and prolonged use of immunosuppressive therapies, patients with SLE face two major risks: a substantially elevated susceptibility to opportunistic infections and an increased incidence of certain malignancies (3, 4). Among these infections, invasive pulmonary aspergillosis (IPA) represents one of the most serious and potentially fatal complications (5). Concurrently, lung cancer, a leading cause of cancer-related morbidity and mortality worldwide, appears to occur more frequently in the SLE population, likely owing to persistent inflammation and impaired immune surveillance (6, 7).

The coexistence of these conditions complicates diagnosis. Structural lung changes resulting from prior infections, such as IPA, may mimic lung cancer on imaging, creating a scenario prone to diagnostic errors. Moreover, cognitive biases, including “anchoring” to a previous infection, can further delay cancer detection. Despite the high prevalence of pulmonary involvement in SLE, comprehensive reports addressing this specific diagnostic pitfall situated at the intersection of SLE, prior IPA, and lung cancer remain scarce.

Here, we present a notable case of a patient with SLE and prior IPA who was ultimately diagnosed with advanced pulmonary squamous cell carcinoma with brain metastases. The aims of this report are threefold: to illuminate the complex “infection-versus-malignancy” diagnostic challenge in this population; to explore the role of cognitive biases in delaying timely diagnosis; and to underscore the importance of systematic re-evaluation when clinical “red flags,” such as treatment failure or atypical disease progression, are observed. This case seeks to enhance clinical awareness and provide practical insights for navigating similarly challenging diagnostic scenarios.

## 2 Case description

A 66-year-old man was admitted with a one-week history of fever and headache. He had a 3-year history of SLE, diagnosed based on the presence of a characteristic malar rash, recurrent high fevers, and high-titer autoantibodies (ANA 1:320, anti-dsDNA > 900 IU/mL). Initial treatment consisted of oral methylprednisolone 8 mg three times daily combined with hydroxychloroquine 0.2 g twice daily. Follow-up after discharge was irregular, and the patient self-adjusted his steroid dosage. A sputum culture identified a fungal infection, supported by significantly elevated serum biomarkers (galactomannan index 3.64 and (1,3)-β-D-glucan 659.6 pg./mL), leading to a diagnosis of IPA. He received voriconazole 200 mg orally twice daily for antifungal therapy for 5 months and had been off medication for over 2 years.

**Figure 1:**
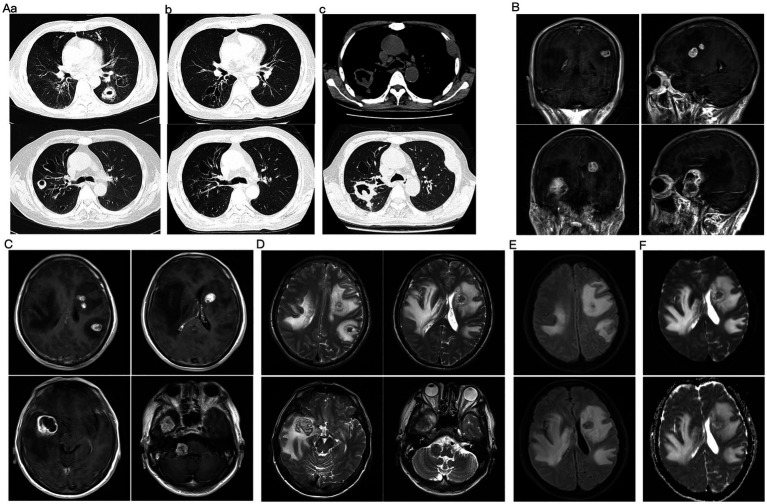
Longitudinal chest CT evolution and brain MRI on current admission. **(A)** Chest CT evolution. (a) Initial CT at IPA diagnosis showing thick-walled cavities in the dorsal segment of the left lower lobe and the anterior segment of the right upper lobe (measuring approximately 35 × 28 mm and 25 × 20 mm), with surrounding infiltrates. (b) Follow-up CT after 4 months of antifungal therapy demonstrating complete resolution of cavities with residual bronchiectasis and fibrotic changes. (c) CT on current admission revealing a new, irregular cavitary mass (approximately 65 × 29 mm) crossing the oblique fissure between the upper and lower lobes of the right lung, distinct from the prior sites of infection. **(B–F)** Brain MRI on current admission. **(B,C)** Post-contrast T1-weighted images showing multiple enhancing nodular lesions in both cerebral hemispheres and the right cerebellum, with significant mass effect and leftward midline shift. The largest lesion, located in the right temporal lobe, measures approximately 27 × 33 mm and demonstrates a lobulated contour. **(D)** T2-weighted image demonstrating the lesions as hypointense with extensive surrounding vasogenic edema. **(E)** FLAIR image highlighting the perilesional edema and mass effect. **(F)** DWI and corresponding ADC map. ADC map showing relatively low signal intensity at the lesion rim, suggesting hypercellularity, while the larger lesion demonstrated central hyperintensity on DWI, consistent with central necrosis.

Upon admission, the patient presented primarily with fever, nocturnal urinary incontinence, and progressive cognitive decline, including memory impairment, slowed responses, and disorientation. Neurological examination suggested central nervous system involvement, revealing mild left upper limb weakness, decreased muscle strength in all limbs (bilateral upper limbs grade IV, bilateral lower limbs grade IV−), diminished tendon reflexes, neck stiffness, and a positive left Babinski sign. Laboratory tests demonstrated moderate anemia (hemoglobin 63 g/L), elevated inflammatory markers (C-reactive protein 68.08 mg/L, erythrocyte sedimentation rate 54 mm/h), and hypocomplementemia (C4 0.17 g/L). Autoantibody testing was consistent with active SLE, showing anti-dsDNA 590.22 IU/mL and positive SSA/Ro-52 and ANA (1:320). Tumor markers were elevated, with cytokeratin fragment 19 (CYFRA21-1) at 17.76 ng/mL and squamous cell carcinoma antigen (SCC) at 5.70 ng/mL. Sputum smear revealed fungal spores and hyphae. Serum galactomannan antigen index was 0.38, and (1,3)-β-D-glucan was <40.00 pg./mL. However, a concurrent sputum culture (specimen collected on day 2) yielded no fungal growth after 2 weeks of incubation. Chest CT revealed an irregular cavitary lesion in the right lung, accompanied by bronchiectasis and mediastinal lymphadenopathy, and incidentally demonstrated a fusiform hypodensity in the left third rib, which was attributed to a benign or non-specific etiology (Figure 1A). Enhanced cranial MRI demonstrated multiple nodular enhancing lesions in both cerebral hemispheres and the right cerebellum, with a leftward midline shift (Figures 1B–F). The initial working diagnosis was intracranial dissemination of IPA, although brain metastases and lupus encephalopathy were considered in the differential. Due to the considerable midline shift on cranial MRI, which posed a significant risk of brain herniation during lumbar puncture (LP), the procedure was postponed.

On hospital day 6, the patient acutely developed severe headache, vomiting, and hypertension (163/90 mmHg). Physical examination revealed horizontal nystagmus and positive bilateral Chaddock’s sign. Electroencephalography demonstrated moderate abnormalities, raising concern for impending cerebral herniation. Following a multidisciplinary discussion, including remote consultation with a neurology expert, it was determined that the potential diagnostic benefit of LP under close monitoring outweighed the risk. The procedure was therefore performed on day 8, revealing an intracranial pressure of 104 mmH_2_O. Cerebrospinal fluid (CSF) analysis showed elevated protein (164.7 mg/dL) and lymphocyte-predominant pleocytosis (50/μL). CSF galactomannan antigen index was 0.11, and (1,3)-β-D-glucan was 47.3 pg./mL. Microbial next-generation sequencing (mNGS) of the CSF detected Aspergillus niger sequences with 30.37% coverage; given its common association with environmental contamination, the clinical significance was uncertain. Aspergillus fumigatus IgG antibody was 68.6 AU/mL (reference <80 AU/mL), and specific IgE was negative. Concurrent serum mNGS identified Aspergillus genus sequences (5.36%), not supporting active aspergillemia, along with Epstein–Barr virus (EBV) DNA.

### 2.1 Therapeutic intervention

Upon admission, given the fever, meningeal signs, and pulmonary cavitary lesion, empirical intravenous moxifloxacin (0.4 g once daily) was initiated for broad-spectrum antimicrobial coverage, along with mannitol for intracranial pressure reduction and supportive care. Given the patient’s history of IPA and the detection of fungal hyphae on sputum smear, there was a strong clinical suspicion of recurrent IPA. Accordingly, oral voriconazole (200 mg every 12 h, following a loading dose on day 1) was added on hospital day 4 for antifungal therapy. Despite this regimen, the patient’s neurological symptoms continued to worsen. To cover possible resistant strains or mixed fungal infections, caspofungin (50 mg intravenously once daily) was introduced on hospital day 7 to broaden the antifungal spectrum. Unfortunately, the patient’s condition still progressed steadily, with deterioration into somnolence by day 11 and coma shortly thereafter.

### 2.2 Diagnostic reassessment and outcome

**Figure 2:**
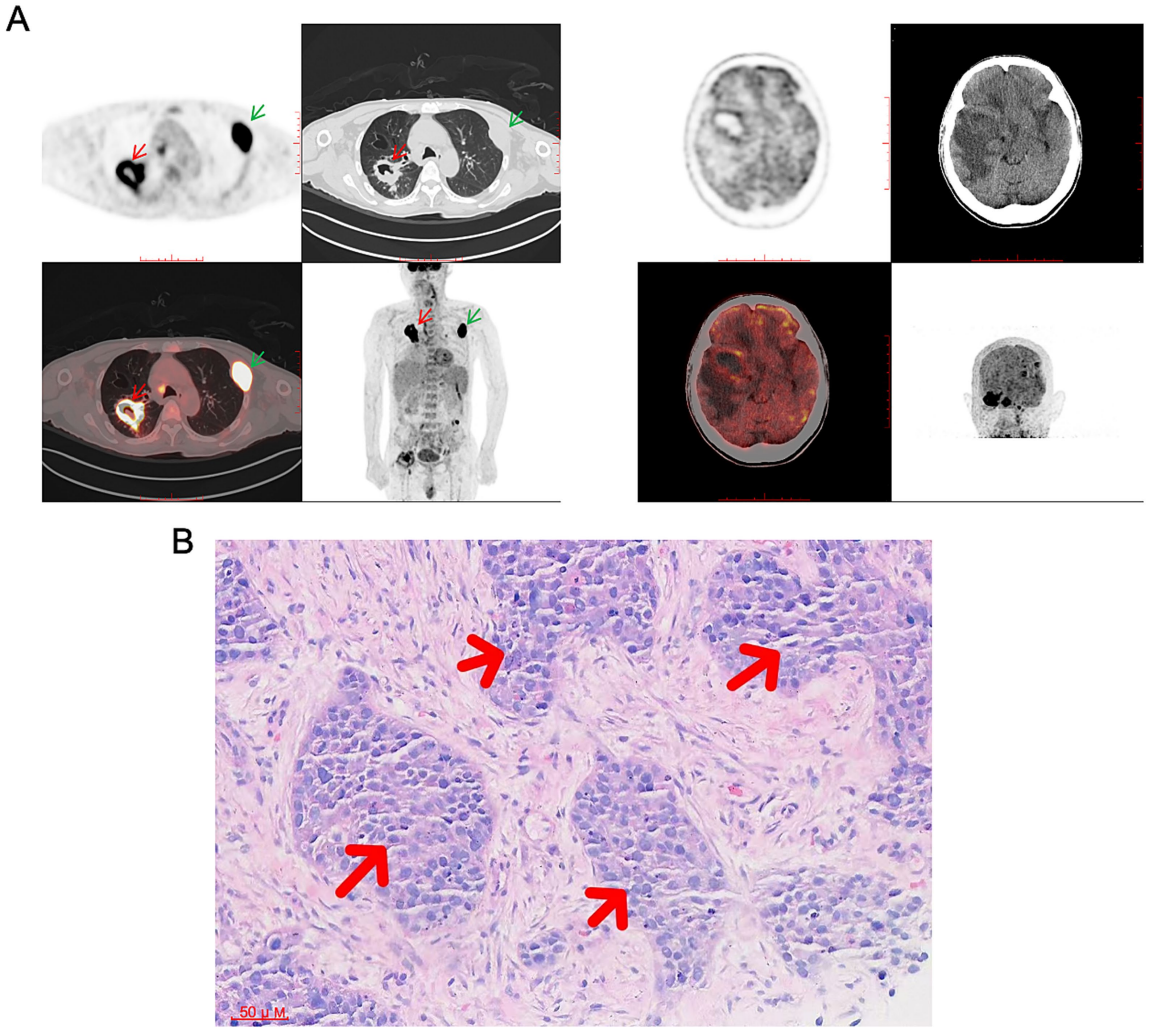
^18^F-FDG PET-CT and histopathological confirmation. **(A)** PET-CT revealed an intensely hypermetabolic cavitary mass (approximately 38 × 40 mm) in the right lung crossing the oblique fissure (red arrow), with widespread metastatic involvement including multiple brain lesions, skeletal metastases (green arrows), and enlarged lymph nodes in bilateral cervical, submandibular, submental, posterior cervical, and supraclavicular regions (largest 9 mm in left submandibular area). **(B)** H&E staining (×200) of the subsequent lung biopsy specimen confirmed the diagnosis, showing nests of malignant cells with pleomorphic nuclei and absent keratinization, consistent with poorly differentiated squamous cell carcinoma.

**Figure 3:**
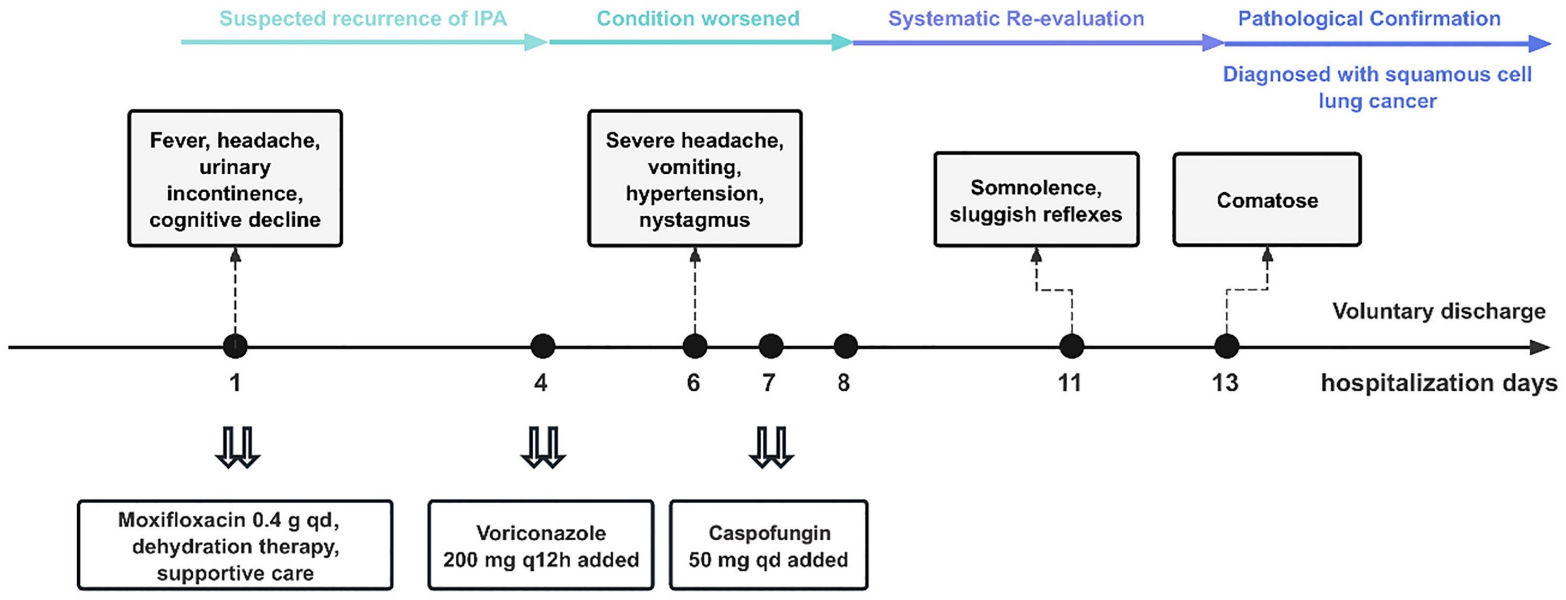
Timeline of diagnosis and treatment.

By day 11, the patient’s mental status deteriorated to somnolence, with sluggish pupillary light reflexes. Given the lack of response to antimicrobial therapy and persistent diagnostic uncertainty, a whole-body ^18^F-FDG PET-CT was performed (Figure 2A). The scan revealed intense metabolic activity in a right pulmonary cavitary mass, multiple intracranial lesions, and multiple skeletal sites, strongly suggestive of metastatic malignancy. Given the extensive tumor burden, extremely poor prognosis, and the patient’s comatose state, the family, after full informed consent, requested voluntary discharge on hospital day 13. After discharge, pathological examination of a lung biopsy specimen (Figure 2B) confirmed poorly differentiated squamous cell carcinoma of the lung (cT4N3M1c, Stage IVB according to the AJCC/UICC TNM Classification, 8th Edition) with brain and bone metastases. By the time of definitive diagnosis, the disease had already reached a terminal stage with extensive metastases, precluding the administration of any anticancer medication and portending an extremely poor prognosis (Figure 3).

## 3 Patient perspective

Direct input from the patient could not be obtained, as he rapidly progressed into a coma following the diagnosis of advanced malignancy. However, based on statements from the family, their experience throughout the diagnostic and therapeutic course evolved from concern about recurrent infection, to confusion over unexplained clinical deterioration, and ultimately to shock at the diagnosis of advanced-stage cancer. The family emphasized that, despite the unfortunate outcome, the timely and definitive diagnosis was crucial for their understanding of the disease and for making subsequent care decisions. They acknowledged the value of publishing this case to highlight such diagnostic challenges for fellow clinicians.

## 4 Discussion

The diagnostic challenge in this case arises from the complex immunopathological interplay among SLE, IPA, and pulmonary squamous cell carcinoma. Substantial evidence indicates that patients with SLE represent a high-risk cohort for lung cancer, with a standardized incidence ratio (SIR) of approximately 1.63 compared to the general population (4). The underlying mechanisms are multifactorial and interrelated. Intrinsic immune dysregulation in SLE, such as persistent activation of the type I interferon pathway and impaired regulatory T cell function, leads to chronic systemic inflammation and immunosuppression (8, 9). This environment facilitates carcinogenic mutations by promoting recurrent alveolar epithelial injury, aberrant tissue repair, and reactive oxygen species (ROS) accumulation (10, 11). Additionally, it diminishes immune surveillance against emerging tumor cells, partly through myeloid-derived suppressor cell (MDSC) expansion (12). Long-term immunosuppressive therapy may further amplify this risk. In the present case, prior IPA infection added substantial complexity. The immunosuppressive milieu inherent to SLE constitutes a shared upstream risk factor for both IPA and lung cancer. Structural lung damage from IPA complicates radiological differentiation, while its immunological sequelae have far-reaching implications. Aspergillus activates the innate immune system via pattern recognition receptors such as Dectin-1 and TLR4, triggering the release of key cytokines including IL-1β, IL-6, and TGF-β (13, 14). These mediators promote Th17 responses and polarize tumor-associated macrophages (TAMs) toward the M2 phenotype, thereby locally establishing a pro-inflammatory, pro-angiogenic, and immunosuppressive microenvironment that supports tumor growth and metastasis (15). It is precisely this vicious cycle and the complex interplay within the “underlying disease-infection-tumor” triad that accounts for the intricate clinical manifestations observed in this patient.

The patient’s significant history of IPA created a strong anchoring bias, leading to a preferential attribution of the new pulmonary and intracranial lesions to recurrent infection. The “fusiform hypodensity” in the left third rib on imaging was dismissed as a non-specific finding based on the presumption of infection, failing to trigger an oncological workup—a classic manifestation of diagnostic inertia driven by anchoring bias (16). The resulting confirmation bias led to the outright interpretation of the fungal hyphae observed on sputum smear as evidence of IPA recurrence, prompting the initiation of voriconazole therapy before culture results were available. This decision failed to account for the potential interference of fungal colonization and overlooked the inhibitory effect of the drug on culture sensitivity. More critically, the absence of CSF and sputum cytology examinations resulted in the systematic omission of early pathological clues that could have directly provided evidence of malignancy. Furthermore, the current Aspergillus biomarkers showed a sharp decline from baseline 2 years prior: serum galactomannan fell from 3.64 to 0.38, and (1,3)-β-D-glucan from 659.6 pg./mL to <40.00 pg./mL—values now well below the threshold for active infection. This argues strongly against recurrent IPA. The low-coverage CSF mNGS sequences were more consistent with colonization or environmental contamination, further supporting that active recurrence was unlikely.

Radiological pitfalls further amplified the diagnostic difficulty. Hematogenous central nervous system (CNS) aspergillosis is relatively uncommon in patients with IPA, with typical imaging features predominantly reflecting vascular invasion, such as hemorrhagic or ischemic infarction (17). However, in immunocompromised hosts, it may also present as multiple microabscesses or granulomas, characteristically located at the corticomedullary junction and appearing as ring-enhancing masses with significant vasogenic edema (18). This “tumor-like” appearance creates substantial radiographic overlap between CNS aspergillosis and intracranial metastatic tumors on conventional MRI (19). In the present case, the initial brain MRI revealed multiple nodular enhancing lesions with surrounding edema—a finding that lacks specificity for differentiating between these two entities. In this context, the dynamic response to treatment became the most critical clinical indicator for breaking the diagnostic deadlock. Effective antifungal therapy at appropriate doses typically leads to clinical stabilization or improvement within 1–2 weeks. However, despite standard antifungal treatment, this patient’s condition progressively worsened. This lack of response strongly challenged the initial diagnosis of “recurrent infection” and necessitated an urgent investigation for an occult malignancy.

To overcome this diagnostic impasse, an institutionalized review pathway is recommended. Specifically, if empirical antimicrobial therapy fails to control disease within a predetermined period (e.g., 7–10 days), a mandatory “Diagnostic Time-out” should be implemented for systematic reassessment. At this critical juncture, PET-CT shifts the diagnostic focus from “distinguishing infection from tumors based on morphology” to “actively identifying highly metabolically active malignancies,” revealing diffuse hypermetabolic activity in lesions. Ultimately, histopathological biopsy remains the gold standard for tumor diagnosis (20, 21). In complex cases unresponsive to adequate antimicrobial therapy, clinical guidelines dictate histopathological confirmation for PET-CT-identified hypermetabolic lesions, constituting an indispensable step for definitive diagnosis and guiding subsequent treatment (22).

Effective management of such complex patients hinges on a systematic pathway from early warning to definitive intervention. The primary prerequisite is the timely recognition of “red flag” signs. For immunocompromised patients complicated by structural lung disease, the following features should prompt immediate tumor screening: persistent clinical and radiographic deterioration despite adequate anti-infective therapy; progressive elevation of tumor markers; or distant metastases unexplained by known infections. Once malignancy is suspected, histopathological biopsy remains the only reliable method for definitive diagnosis and to guide subsequent treatment. All clinical decisions should be grounded in a multidisciplinary team (MDT) approach, integrating expertise from rheumatology, oncology, pulmonology, radiology, and pathology (23). This collaborative model allows for the careful balancing of immunosuppressive therapy and antitumor treatment, facilitating the development of individualized management plans.

Managing SLE patients with advanced lung cancer and CNS metastases demands a delicate balance between immunosuppressive therapy, antitumor treatment, and infection control. Although our patient’s rapid deterioration precluded active treatment, those with better performance status require multidisciplinary guidance. Key considerations include aggressive management of opportunistic infections, judicious corticosteroid use for cerebral edema, palliative radiotherapy for symptomatic metastases, and cautious selection of systemic therapies, particularly immune checkpoint inhibitors, which carry inherent risks of lupus flares in this population (24).

## 5 Conclusion

This case highlights the formidable diagnostic challenge of distinguishing between recurrent infection and a new malignancy in patients with SLE who have a history of structural lung disease and prior invasive fungal infection. A known prior infection can create a strong anchoring bias, contributing to significant diagnostic delays. Clinicians must maintain a high index of suspicion for malignancy when “red flag” signs arise: failure of appropriate antimicrobial therapy; progressive elevation of tumor markers; or the appearance of new lesions suggestive of metastasis. In such complex scenarios, a systematic “diagnostic time-out” should be implemented, during which PET-CT can serve as a pivotal tool to redirect the diagnostic focus toward malignancy. Ultimately, histopathological confirmation remains the diagnostic gold standard. Overcoming cognitive biases, adhering to a structured reassessment pathway, and utilizing an MDT approach are essential to navigate these diagnostic dilemmas and formulate timely, appropriate management strategies for high-risk patients.

## Data Availability

The original contributions presented in the study are included in the article/supplementary material, further inquiries can be directed to the corresponding author.
